# Gonorrhoea and Syphilis Epidemiology in Flemish General Practice 2009–2013: Results from a Registry-based Retrospective Cohort Study Compared with Mandatory Notification

**DOI:** 10.3934/publichealth.2016.4.800

**Published:** 2016-09-27

**Authors:** Christoph Schweikardt, Geert Goderis, Steven Elli, Yves Coppieters

**Affiliations:** 1 Université libre de Bruxelles (ULB), School of Public Health, Research Center 2: Epidemiology, Biostatistics, and Clinical Research; Research Center 3: Health Policies and Systems–International Health, Brussels, Belgium; 2 Catholic University Leuven, Academic Center for General Practice, Leuven, Belgium

**Keywords:** sexually transmitted infections, gonorrhea, syphilis, general practice, morbidity registration, Flanders

## Abstract

**Background:**

The number of newly diagnosed gonorrhoea and syphilis cases has increased in Flanders in recent years. Our aim was to investigate, to which extent these diagnoses were registered by general practitioners (GPs), and to examine opportunities and limits of the Intego database in this regard.

**Methods:**

Data from a retrospective cohort study based on the Flemish Intego general practice database was analyzed for the years 2009–2013. Case definitions were applied. Due to small case numbers obtained, cases were pooled and averaged over the observation period. Frequencies were compared with those calculated from figures of mandatory notification.

**Results:**

A total of 91 gonorrhoea and 23 syphilis cases were registered. The average Intego annual frequency of gonorrhoea cases obtained was 11.9 (95% Poisson confidence interval (CI) 9.6; 14.7) per 100,000 population, and for syphilis 3.0 (CI 1.9; 4.5), respectively, while mandatory notification was calculated at 14.0 (CI: 13.6, 14.4) and 7.0 (CI: 6.7, 7.3), respectively.

**Conclusion:**

In spite of limitations such as small numbers and different case definitions, comparison with mandatory notification suggests that the GP was involved in the large majority of gonorrhoea cases, while the majority of new syphilis cases did not come to the knowledge of the GP.

## 1. Introduction

In the last decade, the number of newly diagnosed sexually transmitted infections (STIs) has risen substantially in Flanders. There, the number of new gonorrhoea cases obtained by mandatory notification increased from 454 in 2006 to a maximum of 1590 in 2015, and the number of syphilis cases from 312 in 2006 to a maximum of 663 cases in 2015, respectively [1].

In 2007, Verhoeven and colleagues deplored the lack of available “reliable data on the importance of the general practice setting in STI diagnoses” [2]. A study on East and West Flanders in 2010 [3] and the follow-up study in 2012–2014 [4] showed that GPs treated nearly 80% of gonorrhoea patients in the study. GPs are the largest group of primary care practitioners, but the extent of their involvement in STI control in Flanders is still incompletely understood. Therefore the aim of this study was (1) to study the quantitative implication of GPs regarding syphilis and gonorrhoea epidemiology in Flanders; and (2) to examine the opportunities and limits of the Intego database for STI surveillance in this regard.

First, the setting will be explained: Aspects of the health system, as far as they are important for surveillance, and characteristics of the major surveillance systems which include STIs will be addressed, before characteristics of the Flemish Intego general practice database are described. After outlining the study design, epidemiological data on syphilis and gonorrhoea are presented in comparative perspective (Intego versus mandatory notification in Flanders) and discussed with regard to the Belgian health system.

### 1.1. The Setting (I): Aspects of the Belgian Health System and STI Surveillance

In Belgium, “compulsory health insurance is combined with a mostly private system of health care delivery, based on independent medical practice, free choice of physician and predominantly fee-for-service payment” ([5], pxxv). Thus, general practitioners (GPs) do not play a role as gatekeepers to specialist care. Diagnosis and treatment of sexually transmitted infections (STIs) are also provided by gynaecologists, dermatologists, urologists, centres for medical students, family planning centres, STI clinics, and AIDS Reference Centres (ARCs) ([6], p16). Furthermore, surveillance of infectious diseases is a regional legal competency. Except for the national HIV cohort, surveyed by the Belgian Scientific Institute of Public Health [7], there is no comprehensive surveillance system for STIs in all Belgium. Instead, there are complementary surveillance systems: First, the Belgian Sentinel Network of Microbiological Laboratories, which includes surveillance of chlamydia trachomatis, syphilis and gonorrhoea ([6], p15). In 2010, 101 microbiological laboratories, or 58% of all, private or hospital-associated, laboratories which were accredited for microbiology, participated voluntarily and unpaid in the network [8]. With regard to syphilis and gonorrhoea, the network has been stable since 2007, covering about 60% of regional and national diagnostic activity ([9], p6). Second, the Belgian STI Sentinel Surveillance Network in cooperation with the Belgian Sentinel Network of General Practices records STI cases including risk determinants and behaviour ([6], pp16-18). Furthermore, in Flanders, gonorrhoea and syphilis are subject to mandatory notification to the Agency for Care and Health [10]. In case of a suspected or proven notifiable disease, treating physicians, physicians who supervise institutions such as enterprises, schools, institutions for children and elderly people, as well as heads of competent laboratories ([11], art45, §3) are obliged to notify the physician in charge of infectious diseases control of their province within 24h [12]. Case definitions for mandatory notification of gonorrhoea ([13], p49) and syphilis ([13], p59) include not only laboratory-confirmed cases but also patients with the clinical picture (clinically suspected, after recent sexual contact with confirmed case). In Flanders, strengthening STI prevention and control constitutes a public health priority [14], and a representative study on sexual health of the Flemish population has been carried out in the so-called “Sexpert” project [15].

### 1.2. The Setting (II): Characteristics of the Intego Database

The Flemish Intego network at the Department of General Practice of the Catholic University Leuven is “the only operational computerized morbidity registration network in Belgium based on general practice data” [16]. Over 90 Intego GPs (see Table 1), evenly spread throughout Flanders, collect data on about 2% “of the Flemish population representative in terms of age and sex” [16] (details see Truyers et al. 2014 [16], Truyers et al. 2015 [17], and Vaes et al. 2015 [18]). The research team at the Academic Centre for General Practice of the Catholic University Leuven coordinates the network and analyses the data. All Intego GPs work with the proprietary software programme Medidoc ®. Every year, all Flemish GPs who work with Medidoc are invited to contribute to the Intego network. Of all GPs responding, quality controls are carried out in order to determine which practices belong to the reference group whose data is used for further analyses [19]. Privacy procedures in place imply that GPs sent their data to a Trusted Third Party (TTP) which assigns codes to the patient identifier and the practice from which the data originates ([20], pp3–4). Therefore the research team at the Academic Centre for General Practice of the Catholic University Leuven does not know from which patient and from which GP the data originates.

A few practices leave the network every year and need to be replaced by new practices, so that the number of practices and GPs slightly fluctuates, but the total number of GPs remained over 90 throughout the observation period ([17], p12). From the yearly contact group, the practice population is calculated by using a correction factor for non-attenders, the group which does not visit their general practitioner in a given period. The correction factor is based on reimbursement data from statutory health insurance, provided by the Intermutualistic Data Agency (IMA-AIM) (details see Bartholomeeusen et al. 2005 [21]). The Intego practice population in the observation period 2009–2013 fluctuated around 150,000, while Flanders had between 6.2 and 6.4 million inhabitants (see Table 1). Omnio status (for households with low income) as a socioeconomic variable had not yet been provided during the observation period [19], so that this data is not available for this study.

Intego GPs routinely register all new diagnoses which are collected together with information on the patient from GPs' personal computers and entered into a central database [18]. GPs are requested to encode clinical labels (keywords) offered by the software programme. To each clinical label, Medidoc assigns a programme-specific internal Medidoc code and a “diagnostic group” code. Furthermore, it links new diagnoses to the International Classiﬁcation of Primary Care (ICPC-2) and the International Statistical Classiﬁcation of Diseases and Related Health Problems, 10th Revision (ICD-10) [18].

Informed consent was not necessary for this type of study. “Intego procedures were approved by the ethical review board of the Medical School of the Catholic University of Leuven (N ML 1723) and by the Belgian Privacy Commission (no SCSZG/13/079)” [18].

## 2. Study Design, Material and Methods

This retrospective cohort study used Intego data of a 5-year time period from 1 January 2009 to 31 December 2013 from the Intego reference group.

The relevant gonorrhoea and syphilis Medidoc codes were selected (see Supplementary Table 1) and cases with these codes included. Two parts of the database were extracted: The first part included the patient numbers, the Medidoc codes, the diagnostic groups gonorrhoea and syphilis, and the start date of the respective diagnosis. The second part included the patient number, the year of birth and sex. The two parts were merged, based on the patient number.

The number of gonorrhoea and syphilis infections was counted as cases (episodes), consisting of one or more patient consultations for the same medical diagnosis. Following the example of Suijkerbuijk and colleagues on chlamydia [22], a second episode with the same diagnosis for the same patient was counted only as a new case after an interval of at least two months after the first diagnosis. We defined the two month-interval as 62 days. Gonorrhoea-syphilis co-infections were defined as registrations of syphilis and gonorrhoea with a beginning date of 7 days or less apart from each other.

One case was excluded due to a software error in which the Medidoc code L09001 for “locomotor congenital syphilis” was assigned to “arm pain not further described”. Six observations with Medidoc code X09131 (“condylomata lata X”) and 11 observations with Medidoc code Y09131 (“condylomata lata Y”) were not included. “Condylomata lata” describes lesions during secondary syphilis [23]. In Medidoc, the diagnostic group “condylomata” includes Medidoc codes for warts of venereal origin and/or ano-genital location including condylomata acuminata and condylomata lata. For these 17 patients, no diagnostic code for syphilis was registered during the observation period. For comparison, the Agency for Care and Health provided data on annual syphilis and gonorrhoea cases obtained by mandatory notification, differentiated by age and sex.

## 3. Data Analysis

Descriptive analysis was performed with STATA 12.0 and Excel 2010. For gonorrhoea and syphilis, the number of cases per year was calculated and Poisson confidence intervals (CIs) applied. For privacy reasons, only the year of birth was given in the Intego database. In order to estimate the age class, July 1 was assigned as fictitious birthday to all patients in order to keep the error of the estimated age within the range of half a year or less. Then the estimated age was calculated by subtracting the fictitious birthday from the beginning date of the diagnosis. Four age classes were chosen for comparison with mandatory notification data.

Taking the age group 45+ years as reference group; odds ratios (ORs) with regard to the other age groups were calculated. For age groups which have fewer than 5 cases, no OR is presented due to the lack of precision.

Due to small numbers obtained, the annual numbers of syphilis and gonorrhoea cases were pooled and averaged for 2009–2013 and the frequency per 100,000 practice population calculated. For comparison, the annual gonorrhoea and syphilis frequencies in the Flemish population were calculated from the figures of the Agency for Care and Health, Flanders ([1] and unpublished data), and population statistics taken from the Belgian Federal Public Service Economy [24]. 95% Poisson confidence intervals were applied as well.

## 4. Results

During the observation period 2009–2013, 91 gonorrhoea were registered in the Intego database (see Table 1). For one Intego patient, two gonorrhoea cases were registered, one in November 2012 and one in April 2013. Between 15 and 22 gonorrhoea episodes annually without a clear trend were reported by Intego GPs. No Intego patient was registered with both syphilis and gonorrhoea. Mandatory reporting obtained 622 gonorrhoea cases in 2009 and 1162 cases in 2013 with a clear upwards trend (see Table 1).

Furthermore, Intego GPs reported 23 syphilis cases with a decline of 7 syphilis episodes in 2009 to two in 2013. Mandatory reporting of syphilis showed no clear trend with a maximum of 520 syphilis cases in 2009 and a minimum of 385 cases in 2010 (see Table 1).

**Table 1.** Number of registered annual syphilis and gonorrhoea cases, Flanders 2009-2013, Intego database and mandatory notification.

Year		2009	2010	2011	2012	2013	Total over 5 years
Number of Intego physicians		111	116	109	105	98	
Intego patient population		159698	156068	166740	137796	141631	
Male Intego practice population		80329	78240	83470	68586	71049	
Female Intego practice population		79369	77828	83270	69210	70582	
Number of Intego gonorrhoea cases	Total	15	22	17	21	16	**91**
	Males	14	20	15	16	11	**76**
	Females	1	2	2	5	5	**15**
Number of Intego syphilis cases	Total	7	6	4	4	2	**23**
	Males	5	5	3	4	1	**18**
	Females	2	1	1	0	1	**5**
Flanders' population (rounded)		6230430	6279311	6328702	6366312	6396282	
Male Flanders' population (rounded)		3074752	3099352	3124461	3143509	3158593	
Female Flanders' population (rounded)		3155679	3179959	3204241	3222803	3237690	
Number of gonorrhoea cases, mandatory notification	Total	622	763	918	958	1162	**4423**
	Males	517	601	727	765	943	**3553**
	Females	87	139	167	183	219	**795**
	Sex unknown	18	23	24	10	0	**75**
Number of syphilis cases, mandatory notification	Total	520	385	469	391	447	**2212**
	Males	443	308	381	335	376	**1843**
	Females	68	64	77	54	69	**332**
	Sex unknown	9	13	11	2	2	**37**

Sources: Practice population: Intego database, yearly practice population calculated from yearly contact group. Flanders' population: annual mean population (sum of the number of January 1 of the year of reference and of January 1 of the following year), divided by 2, rounded. Source: FOD Economie—ADSEI. Processing: Research Service of the Flemish Government (Studiedienst van de Vlaamse Regering (SVR)). Numbers of gonorhoea and syphilis cases: Intego database and Agency for Care and Health, Flanders (mandatory notification).

### 4.1. Annual Number of Gonorrhoea and Syphilis Cases Registered per 100,000 Population

**Figure 1.**
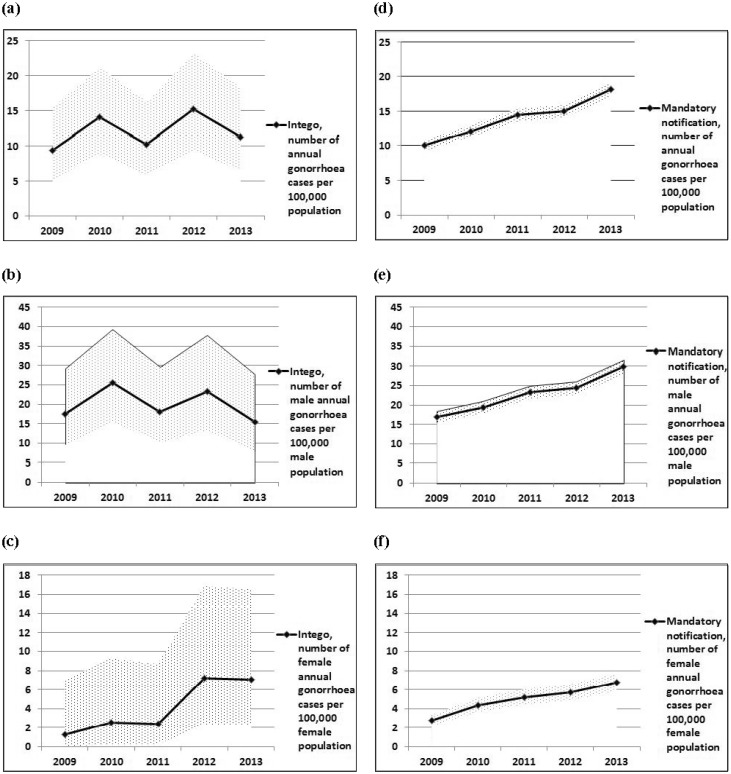
Annual number of gonorrhoea cases per 100,000 population, Intego database, Flanders, in comparison with mandatory notification 2009–2013 (1a–c: Intego; 1d–f: Mandatory notification). Reference population: Intego: Practice population (PP), mandatory notification: Flanders' population (FP). FP: annual mean population (sum of the number of January 1 of the year of reference and of January 1 of the following year, divided by 2, rounded). Sources: PP: Intego database, yearly practice population calculated from yearly contact group. FP: FOD Economie—ADSEI. Processing: Research Service of the Flemish Government (Studiedienst van de Vlaamse Regering (SVR)). Annual frequencies calculated from gonorrhoea numbers. Sources for gonorrhoea numbers: Intego database and Agency for Care and Health, Flanders (mandatory notification). 95% Poisson confidence interval shaded. Mandatory notification: sex unknown for 18 cases in 2009, 23 cases in 2010, 24 cases in 2011, 10 cases in 2012, and 0 cases in 2013.

In the Intego database, the annual number of gonorrhoea cases per 100,000 practice population in 2009–2013 varied between 9.4 and 15.2 in total without a clear upwards trend, between 15.5 and 25.6 in men, and between 1.3 and 7.2 in women, respectively (see Figure 1a–c). Mandatory notification showed an increase from 10.0 in 2009 to 18.2 in 2013 in total, with an increase from 16.8 to 29.9 in men and 2.8 to 6.8 in women (see Figure 1d–f).

**Figure 2.**
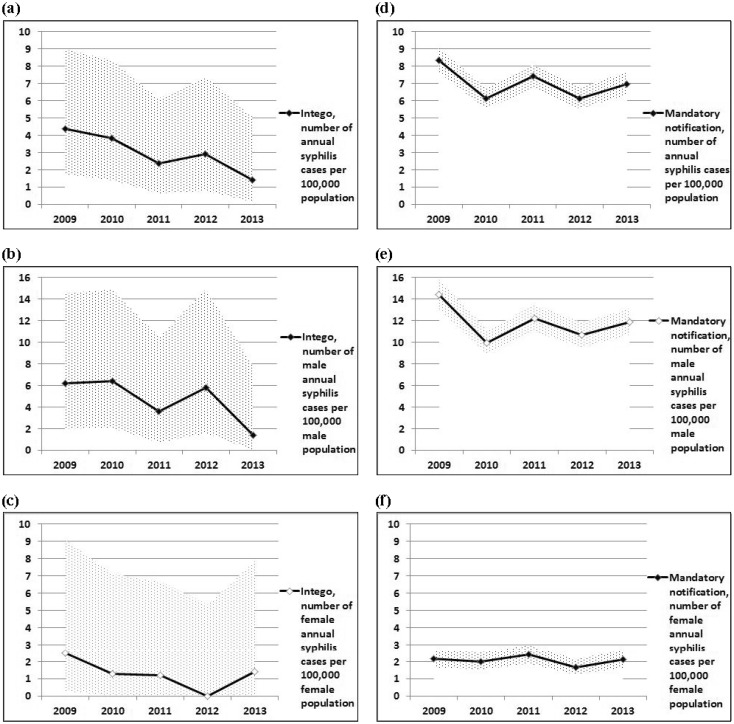
Annual number of syphilis cases per 100,000 population, Intego database, Flanders, in comparison with mandatory notification 2009–2013 (2a–c: Intego; 2d–f: Mandatory notification). Reference population: Intego: Practice population (PP), mandatory notification: Flanders' population (FP). FP: annual mean population (sum of the number of January 1 of the year of reference and of January 1 of the following year, divided by 2, rounded). Sources: PP: Intego database, yearly practice population calculated from yearly contact group. FP: FOD Economie—ADSEI. Processing: Research Service of the Flemish Government (Studiedienst van de Vlaamse Regering (SVR)). Annual frequencies calculated from gonorrhoea numbers. Sources for gonorrhoea numbers: Intego database and Agency for Care and Health, Flanders (mandatory notification). 95% Poisson confidence interval shaded (Figure 2c: 2012 97.5% upper limit shaded since point estimation was 0). Mandatory notification: sex unknown for 9 cases in 2009, 13 cases in 2010, 11 cases in 2011, 2 cases in 2012, and 2 cases in 2013.

For syphilis, the point estimation for the annual frequency per population in the Intego database decreased from 4.4 to 1.4 per 100,000 population with a maximum of 6.4 and a minimum of 1.4 in men and 2.5 and 0 in women, respectively, while mandatory notification showed a varying picture between 8.4 and 6.1 without a clear trend, with a maximum of 14.4 and a minimum of 9.9 in men as well as 2.4 and 1.7 in women, respectively (see Figure 2d–f).

For both gonorrhoea and syphilis, the small number of Intego cases corresponds with large confidence intervals in comparison with mandatory notification, so that trends in the Intego database cannot be established with certainty.

### 4.2. Sex and Age Distribution

In both databases, the majority of male gonorrhoea patients were 25 to 44 years old. Men accounted for 76 of 91 (84%) of Intego gonorrhoea cases, a male to female ratio of 5.1, while mandatory notification showed a ratio of 4.5. More than one fifth of total gonorrhoea cases in both databases were 45 years of age or older. The ORs for age groups 15–24 and 25–44 years compared with the reference group of 45 years and older—between 3 and 4 for Intego and between 3 and 5 for mandatory notification—are similar for total gonorrhoea numbers and for male gonorrhoea in both databases. The small female gonorrhoea numbers in the Intego database are associated with large OR confidence intervals. Mandatory notification shows higher odds for females than for males in the age groups 15–24 years and 25–44 years compared with the reference group of 45 years and older (see Table 2a–c).

In both databases, the majority of syphilis patients, males and females, belonged to the age group 25–44 years, and the odds of males in the age group 25–44 years to be diagnosed with syphilis was higher than in the reference group of 45 years and older. Due to small numbers, the OR is not significant for male syphilis cases in the Intego database. Female Intego syphilis numbers are too small for calculating meaningful ORs (see Table 3a–c).

**Table 2a–c:** Number of gonorrhoea cases by age and sex including Odds Ratios (ORs) with persons of 45 years and older as reference group, Flanders 2009–2013, Intego database (n = 91), compared with mandatory notification cases of which the sex is known (n = 4348).

**Table 2a:** Total number of gonorrhoea cases, Flanders, 2009–2013, Intego database (n = 91) and mandatory notification (n = 4348), per age group.

Intego database								Mandatory notification						
Age (years)	Total (n)	% of total (rounded)	Ref. pop. (rounded)	OR	95% CI LL	95% CI UL	*p*	Total (n)	% of total (rounded)	Ref. pop. (rounded)	OR	95% CI LL	95% CI UL	*p*
0–14	0	0	129146	n.a.	n.a.	n.a.	n.a.	33	1	5106016	0.11	0.08	0.15	<0.001
15–24	23	25	98550	3.52	1.97	6.32	<0.001	1002	23	3687677	4.53	4.14	4.96	<0.001
25–44	47	52	217176	3.27	1.96	5.44	<0.001	2358	55	8225274	4.78	4.42	5.16	<0.001
45+	21	23	317061	1.00				875	21	14582070	1.00			
Age unknown	0	0	n.a.	n.a.	n.a.	n.a.	n.a.	80	2	n.a.	n.a.	n.a.	n.a.	n.a.
**All ages**	**91**	**100**	**761933**					**4348**	**100**	**31601036**

**Table 2b.** Male gonorrhoea cases, Flanders, 2009–2013, Intego database (n = 76) and mandatory notification (n = 3553), per age group.

Intego database								Mandatory notification						
Age (years)	Total (n)	% of total (rounded)	Ref. pop. (rounded)	OR	95% CI LL	95% CI UL	*p*	Total (n)	% of total (rounded)	Ref. pop. (rounded)	OR	95% CI LL	95% CI UL	*p*
0–14	0	0	67076	n.a.	n.a.	n.a.	n.a.	21	1	2610597	0.07	0.05	0.11	<0.001
15–24	17	22	49811	3.27	1.67	6.39	<0.001	733	21	1873972	3.58	3.24	3.96	<0.001
25–44	43	57	111440	3.70	2.10	6.52	<0.001	1972	56	4151499	4.35	4.00	4.73	<0.001
45+	16	21	153347	1.00				761	21	6964598	1.00			
Age unknown	0	0	n.a.	n.a.	n.a.	n.a.	n.a.	66	2	n.a.	n.a.	n.a.	n.a.	n.a.
**All ages**	**76**	**100**	**381674**					**3553**	**100**	**15600665**

**Table 2c.** Female gonorrhoea cases, Flanders, 2009-2013, Intego database (n = 15) and mandatory notification (n = 795), per age group.

Intego database								Mandatory notification						
Age (years)	Total (n)	% of total (rounded)	Ref. pop. (rounded)	OR	95% CI LL	95% CI UL	*p*	Total (n)	% of total (rounded)	Ref. pop. (rounded)	OR	95% CI LL	95% CI UL	*p*
0–14	0	0	62070	n.a.	n.a.	n.a.	n.a.	12	2	2495420	0.32	0.18	0.58	<0.001
15–24	6	40	48739	4.03	1.31	12.43	0.023	269	34	1813705	9.91	7.97	12.33	<0.001
25–44	4	27	105736	n.a.	n.a.	n.a.	n.a.	386	49	4073775	6.33	5.14	7.80	<0.001
45+	5	33	163714	1.00				114	14	7617472	1.00			
Age unknown	0	0	n.a.	n.a.	n.a.	n.a.	n.a.	14	2	n.a.	n.a.	n.a.	n.a.	n.a.
**All ages**	**15**	**100**	**380259**					**795**	**100**	**16000371**				

Ref.pop: reference population. Intego reference population: Sum of calculated annual Intego patient populations 2009–2013. Source: Intego database, yearly practice population calculated from yearly contact group. Flemish reference population: sum of annual mean populations (sum of the number of January 1 of the year of reference and of January 1 of the following year, divided by 2), Flanders 2009–2013. Source: FOD Economie—ADSEI. Processing: Research Service of the Flemish Government (Studiedienst van de Vlaamse Regering (SVR)). Numbers of gonorhoea and syphilis: Sources: Intego database and Agency for Care and Health, Flanders (mandatory notification). OR: odds ratio, CI: confidence interval, LL: lower limit, UL: upper limit. Reference group for calculation of OR: age group 45+. Not listed: 75 gonorrhoea cases of unknown sex (mandatory notification). Comparison with reference group: no OR is given for cells with <5 cases; n.a.: not available.

**Tables 3a–c.** Number of syphilis cases by age and sex including Odds Ratios (ORs) with persons of 45 years and older as reference group, Flanders 2009–2013, Intego database (n = 23), compared with mandatory notification cases of which the sex is known (n = 2175).

**Table 3a:** Total number of syphilis cases, Flanders, 2009–2013, Intego database (n = 23) and mandatory notification (n = 2175), per age group.

Intego database								Mandatory notification						
Age (years)	Total (n)	% of total (rounded)	Ref. pop. (rounded)	OR	95% CI LL	95% CI UL	*p*	Total (n)	% of total (rounded)	Ref. pop. (rounded)	OR	95% CI LL	95% CI UL	*p*
0–14	0	0	129146	n.a.	n.a.	n.a.	n.a.	19	1	5106016	0.09	0.05	0.13	<0.001
15–24	1	4	98550	n.a.	n.a.	n.a.	n.a.	258	12	3687677	1.60	1.39	1.85	<0.001
25–44	14	61	217176	2.55	1.10	5.94	0.03	1217	56	8225274	3.39	3.08	3.73	<0.001
45+	8	35	317061	1.00				636	29	14582070	1.00			
Age unknown	0	0	n.a.	n.a.	n.a.	n.a.	n.a.	45	2	n.a.	n.a.	n.a.	n.a.	n.a.
**All ages**	**23**	**100**	**761933**					**2175**	**100**	**31601036**

**Table 3b.** Male syphilis cases, Flanders, 2009-2013, Intego database (n = 18) and mandatory notification (n=1843), per age group.

Intego database								Mandatory notification						
Age (years)	Total (n)	% of total (rounded)	Ref. pop. (rounded)	OR	95% CI LL	95% CI UL	*p*	Total (n)	% of total (rounded)	Ref. pop.(rounded)	OR	95% CI LL	95% CI UL	*p*
0–14	0	0	67076	n.a.	n.a.	n.a.	n.a.	13	1	2610597	0.06	0.04	0.10	<0.001
15–24	1	6	49811	n.a.	n.a.	n.a.	n.a.	176	10	1873972	1.14	0.96	1.35	0.130
25–44	11	61	111440	2.52	0.97	6.57	0.08	1044	57	4151499	3.06	2.76	3.38	<0.001
45+	6	33	153347	1.00				573	31	6964598	1.00			
Age unknown	0	0	n.a.	n.a.	n.a.	n.a.	n.a.	37	2	n.a.	n.a.	n.a.	n.a.	n.a.
**All ages**	**18**	**100**	**381674**					**1843**	**100**	**15600665**

**Table 3c.** Female syphilis cases, Flanders, 2009–2013, Intego database (n = 5) and mandatory notification (n = 332), per age group.

Intego								Mandatory notification						
Age (years)	Total (n)	% of total (rounded)	Ref. pop. (rounded)	OR	95% CI LL	95% CI UL	*p*	Total (n)	% of total (rounded)	Ref. pop. (rounded)	OR	95% CI LL	95% CI UL	*p*
0–14	0	0	62070	n.a.	n.a.	n.a.	n.a.	6	2	2495420	0.29	0.13	0.66	0.001
15–24	0	0	48739	n.a.	n.a.	n.a.	n.a.	82	25	1813705	5.47	3.94	7.58	<0.001
25–44	3	60	105736	n.a.	n.a.	n.a.	n.a.	173	52	4073775	5.13	3.85	6.84	<0.001
45+	2	40	163714	1.00				63	19	7617472	1.00			
Age unknown	0	0	n.a.	n.a.	n.a.	n.a.	n.a.	8	2	n.a.	n.a.	n.a.	n.a.	n.a.
**All ages**	**5**	**100**	**380259**					**332**	**100**	**16000371**				

Ref.pop: reference population. Intego reference population: Sum of calculated annual Intego patient populations 2009–2013. Source: Intego database, yearly practice population calculated from yearly contact group. Flemish reference population: sum of annual mean populations (sum of the number of January 1 of the year of reference and of January 1 of the following year, divided by 2), Flanders 2009–2013. Source: FOD Economie—ADSEI. Processing: Research Service of the Flemish Government (Studiedienst van de Vlaamse Regering (SVR)). Numbers of gonorhoea and syphilis: Sources: Intego database and Agency for Care and Health, Flanders (mandatory notification). OR: odds ratio, CI: confidence interval, LL: lower limit, UL: upper limit. Reference group for calculation of OR: age group 45+. Not listed: 37 syphilis cases of unknown sex (mandatory notification). Comparison with reference group: no OR is given for cells with <5 cases; n.a.: not available.

### 4.3. Annual Gonorrhoea and Syphilis Cases Averaged 2009–2013 per 100,000 Population, Flanders, Intego Database and Mandatory Notification

In the Intego database, the average annual gonorrhoea frequency per practice population for the 5-year-period 2009–2013 was 11.9 per 100,000 population, with 19.9 for men and 3.9 for women. For syphilis, the average annual frequency per practice population represented 3.0 per 100,000 population, with 4.7 for men and 1.3 for women. Mandatory notification figures resulted in a point estimation of 14.0 for gonorrhoea, with 22.8 for men and 5.0 for women, respectively, and for syphilis of 7.0, with 11.8 for men and 2.1 for women, respectively (see Table 4).

**Table 4.** Annual gonorrhoea and syphilis cases averaged 2009–2013 per 100,000 reference population, Flanders, Intego database and mandatory notification.

	Intego database					Mandatory notification				
	n	av PP	av Freq	LL	UL	n	av FP	av Freq	LL	UL
**Gonorrhoea total**	91	152387	11.9	9.6	14.7	4423	6320207	14.0	13.6	14.4
Men	76	76335	19.9	15.7	24.9	3553	3120133	22.8	22.0	23.5
Women	15	76052	3.9	2.2	6.5	795	3200074	5.0	4.6	5.3
Sex unknown	0	n.a.	n.a.	n.a.	n.a.	75	n.a.	n.a.	n.a.	n.a.
**Syphilis total**	23	152387	3.0	1.9	4.5	2212	6320207	7.0	6.7	7.3
Men	18	76335	4.7	2.8	7.5	1843	3120133	11.8	11.3	12.4
Women	5	76052	1.3	0.4	3.1	332	3200074	2.1	1.9	2.3
Sex unknown	0	n.a.	n.a.	n.a.	n.a.	37	n.a.	n.a.	n.a.	n.a.

n: number of cases, LL/UL: 95% Poisson confidence interval, lower/upper limit (rounded), av PP: average practice population (rounded), av. FP: average Flanders' population (rounded), av Freq: average number of cases per year and 100,000 practice population (Intego), average number of cases per year and 100,000 Flanders' population (mandatory notification); n.a.: not available.

## 5. Discussion

This retrospective descriptive study gives new insight into the extent of GP involvement regarding gonorrhoea and syphilis in Flanders as well as the opportunities and limits of the Intego database. Strengths of this study are that the data was collected as part of routine activities as Intego GPs. No additional efforts of GPs were necessary to obtain the data, and there was no special focus on STIs, thus reducing possible bias.

Verbrugge and colleagues interpret the annually reported syphilis and gonorrhoea cases in the Sentinel Network of Microbiological Laboratories per reference population as reported incidence [6],[9]. We also interpret annual Intego and mandatory notification figures per year and reference population as reported incidence, since new cases came to the knowledge of and were registered by Intego GPs and the Agency for Care and Health, respectively, whether or not these cases constituted new infections or newly discovered but previously acquired ones.

The interpretation of the data requires caution. The study situates itself within the context of incomplete figures concerning Belgian STI surveillance and a substantial variability between different European countries [25]. The data confirm the results of Laisnez et al., who found that the GP in East and West Flanders treated gonorrhoea in the large majority of cases [3],[4], as well as the dilemma that most gonorrhoea infections are seen by the GP but that it is a rare disease for the GP and a challenge to keep him/her up-to-date with the latest relevant guidelines [3]. Comparison with mandatory notification suggests that the majority of new syphilis cases did not come to the knowledge of the GP.

We do not know the exact extent of under-reporting to mandatory notification of syphilis and gonorrhoea in Flanders. At least for gonorrhoea, the comparison of mandatory notification figures with the figures from the Sentinel Network of Microbiological Laboratories might give an indication. The number of gonorrhoea cases in Flanders registered by the Sentinel Network of Microbiological Laboratories rose from 368 in 2009 to 600 in 2013 ([9], p21). If 60% regional coverage is assumed ([9], p6), the point estimations for the annual number of cases diagnosed among the Flemish population remain below the number of cases obtained by mandatory notification. Even if the case definitions are different—mandatory notification includes also clinical (suspected) cases, there seems to be not very much under-reporting of gonorrhoea in Flanders. Regarding syphilis, the situation is more difficult. Kenyon and colleagues pointed out the challenges to diagnose syphilis reinfection [26], and the Belgian Scientific Institute of Public Health referred to the uncertainty as to whether the annual syphilis cases reported by the Belgian Sentinel Network of Microbiological Laboratories concerned ancient treated or recent infections ([6], p39). The number of syphilis cases obtained from the Sentinel Network of Microbiological Laboratories during the observation period ranged between a minimum of 428 cases in 2010 and a maximum of 732 cases in 2013 ([9], p23) with annual numbers for men being higher (between 394 and 659) and for women being lower (between 33 and 65) than those obtained by mandatory notification throughout the observation period. We do not know to which extent under-reporting and suspected but not confirmed clinical cases counterbalanced each other in mandatory notification. The report of the Belgian Scientific Institute of Public Health for 2011 demonstrates that in French-speaking Belgium substantial mandatory notification under-reporting of syphilis and gonorrhoea occurred, when compared with the figures from the Sentinel Network of Microbiological Laboratories (see [27], pp34–35, pp46–47). By 2012, mandatory notification of gonorrhoea and syphilis (except congenital syphilis) was abolished in Wallonia ([28], p16). This means that a patient residing in Flanders and being diagnosed in Wallonia will, in most cases, not be notified and thus not be entered into the Flemish mandatory notification database (personal information from Valeska Laisnez, infectious diseases control, Agency for Care and Health, Flanders, 04.08.2016).

Nevertheless, Flemish mandatory notification gonorrhoea figures seem closest to the number of annually diagnosed cases, and if there is substantial under-reporting of syphilis to mandatory notification, then the implication of Intego GPs is even smaller than expressed in comparison with mandatory notification.

Due to privacy protection procedures, the contribution of individual Intego GPs to the overall figures in the database is not known. This means that there is no possibility to match registrations from Intego GPs with notifications to the Flemish Agency for Care and Health, or to locate the cases exactly geographically within Flanders. Furthermore, a possibly skewed distribution (a subset of GPs providing virtually all STI diagnoses and the others none) cannot be traced in the Intego database. According to the Belgian Sentinel Network of General Practices, to which 146 Belgian sentinel GP practices reported in 2013, half of them reported no STI (of four STIs investigated, namely chlamydia trachomatis, syphilis, gonorrhoea and genital warts (condylomata acuminata)) in that year ([6], p47). As a mean, one STI was reported by sentinel GP practices in 2013 ([6], pp47–48).

While Belgium did not report the transmission category, the European Centre for Disease Prevention and Control (ECDC) showed that as a mean 43% of gonorrhoea cases ([25], p18) and 58% of syphilis cases ([25], p28) in countries of the European Union/European Economic Area with known transmission in 2013 (not all countries included in the statistics) were men who had sexual relations with men (MSM).

The STI Sentinel Surveillance Network found that in 2013 88% of men with syphilis and 84% of men with gonorrhoea were MSM ([6], p57). A study on people attending the HIV/STI clinic at the Institute of Tropical Medicine in Antwerp from 1992 to 2012 found that syphilis “was only diagnosed in persons who were HIV positive at the time of the diagnosis or who became HIV positive at a later date” ([26], p2). A voluntary outreach counselling and testing study on STIs/HIV in Antwerp found that “[i]nfected MSM were significantly less often registered with a fixed general practitioner” ([29], p172). In a low-threshold HIV testing project in French-speaking Belgium, 28% of MSM reported not having a regular GP ([30], p11). These and further findings from a STI/HIV help centre in Brussels ([31], slide 13) suggest that, in particular, syphilis cases of a sub-group of MSM preferring anonymity, concealing their sexual orientation vis-à-vis their GP, deliberately not consulting their GP or not having a GP at all, might be under-registered in general practice. Thus, the difference in reported incidence of male syphilis between the Intego database and mandatory notification might largely be attributed to this subgroup of MSM.

### 5.1. Limitations of the Study

#### 5.1.1. Difference of Case Definitions

Comparison of annual Flemish case numbers from different data sources needs to be carried out prudently because the case definitions are different. As stated above, mandatory notification is based on suspected (clinical) or confirmed cases. In Intego, it is the judgement of the physician to register a code signifying gonorrhoea or syphilis, whatever the justification (laboratory result, microscopy, clinical picture, report, or other).

#### 5.1.2. Completeness of GP Registration and Extent of Specialist Reporting

Jamoulle pointed out that it is generally not known how complete electronic health records of GPs are regarding the health information of their patients ([32], p19). It is not known to which extent GPs did not get reports from other healthcare providers or did not register them, and to which extent STI patients did not have a GP or did not wish to have their GP informed by the specialist. To be on the safe side, we can state that all data in the electronic health record reported to Intego came to the knowledge to the GP, meaning he/she was involved either based on his/her own diagnosis or on information from other healthcare providers.

#### 5.1.3. Challenge of Transferability to all Flanders

While the practice population is representative for the Flemish population, we do not know whether STI investigation strategies and communication patterns with other healthcare providers of Intego GPs are representative for Flemish GPs as well. It is likely that Intego GPs are more than average committed to their profession. If their STI detection strategies are better than average, then the annual Intego figures per 100,000 reference population might be higher than those obtained from GPs in all Flanders.

#### 5.1.4. Small Annual Numbers

The small number of cases registered annually inevitably led to wide confidence intervals, signaling limited reliability and precision. The annually visible trend of increasing numbers of gonorrhoea diagnoses between 2009 and 2013, reported from the Belgian Sentinel Network of Laboratories and from mandatory notification in Flanders, is not visible in the Intego database.

In their study on cancer registries, Takiar and colleagues argued “that when the population of the registry is around 150,000 then the incidence rate of even 10 should be viewed with reservations” ([33], p660). The patient population of the Intego database has about this order of magnitude.

### 5.2. Opportunities for Further Research

Further research could look into ways to improve the cooperation between the GP and the specialist on STIs/HIV, as recommended by the Belgian HIV Plan 2014–2019: “A system should be developed that allows the bi-and multi-directional exchange of data (including drug therapy data) on the GMR [Global Medical Record] between the general practitioner and the specialist(s)” ([34], p28).

Simple adjustments to the collection of Intego data could lead to further insights. (1) The possibility to mark a diagnosis as “reported” by another healthcare provider would show the extent of other health care providers contributing to diagnoses in general practice. Furthermore, reporting culture of specialists could be examined. (2) Assigning the criterion of a period of more than two months between two registrations of the same diagnosis in order to distinguish one episode of care or one case from another is necessarily arbitrary. The “Weekly Returns Service”, today under the name Royal College of General Practice Research and Surveillance Centre (RCGP-RSC) [35], a general practice network serving England, counts episodes as “first” (first diagnosis ever), “new” (new episode of care) and “ongoing” (another registration during an episode of care having started previously) ([36], p88). Taking over these categories could add precision to case definitions in the network.

## 6. Conclusion

The Intego database offers epidemiological data on selected STIs from routine registration in general practice in Flanders, giving an indication on quantitative GP involvement in STI control. Although reliability and precision are limited by small numbers, especially for syphilis, comparison with mandatory notification suggests that GPs came to know the large majority of gonorrhoea episodes, but not the majority of syphilis episodes, for which reporting from specialist care seems not very developed. Opportunities for further development of the Intego database include discriminating GP-diagnosed episodes from episodes registered there but diagnosed by other healthcare providers.

## Figures and Tables

**Table 1.** Gonorrhoea and syphilis diagnostic group codes, Medidoc codes, and Medidoc clinical label in Dutch selected for extraction, the latter with English equivalent.

Diagnostic group code (English equivalent)	Medidoc internal code	Medidoc clinical label (keyword)	English equivalent of Medidoc clinical label
**GONORRHOEA**			
GONORRHOEA	AA549	Gonorroe	Gonorrhoea
GONORRHOEA	AA5491	Gonorroe	Gonorrhoea
GONORRHOEA	AA5492	Infectie door Neisseria gonorrhoeae	Neisseria gonorrhoeae infection
GONORRHOEA	AA5494	Gonorroe	Gonorrhoea
GONORRHOEA	AA5498	Gonorroe	Gonorrhoea
GONORRHOEA	AA5499	Gonorroe	Gonorrhoea
GONORRHOEA	B09889	Gonokokkemie	Gonococcaemia
GONORRHOEA	DA546	Gonokokken rectitis	Gonococcal proctitis
GONORRHOEA	DA5461	Gonokokken rectitis	Gonococcal proctitis
GONORRHOEA	HA545	Farynx infectie door gonokokken	Gonococcal pharynx infection
GONORRHOEA	KI3201	Pericarditis akuut infektieus bacterieel gonokokkaal	Acute infectious bacterial gonococcal pericarditis
GONORRHOEA	KI3981	Endocarditis gonokokken	Gonococcal endocarditis
GONORRHOEA	KI4102	Myocarditis gonokokken	Gonococcal myocarditis
GONORRHOEA	L0985	Artritis gonorroeisch	Gonorrhoeal arthritis
GONORRHOEA	LM730	Bursitis door gonokokken	Gonococcal bursitis
GONORRHOEA	MDC_X71	Gonorroe vrouw	Female gonorrhoea
GONORRHOEA	MDC_Y71	Gonorroe man	Male gonorrhoea
GONORRHOEA	U0980	Infektie gonokokken urethra akuut	Acute gonococcal urethra infection
GONORRHOEA	U09801	Urethritis gonorroe akuut	Acute gonorrhoeal urethritis
GONORRHOEA	U0982	Infektie gonokokken urethra chronisch	Chronic gonococcal urethra infection
GONORRHOEA	U583811	Nefritis intersititieel + gonokokken infektie	Interstitial nephritis and gonococcal infection
GONORRHOEA	UA5403	Gonokokken urethritis	Gonococcal urethritis
GONORRHOEA	UA54031	Urethritis gonokokken	Gonococcal urethritis
GONORRHOEA	UA54039	Gonorroe urethritis	Gonorrhoeal urethritis
GONORRHOEA	UN2911	Gonokokken ureteritis	Gonococcal ureteritis
GONORRHOEA	UY5403	Urethritis door gonokokken Y	Male gonococcal urethritis
GONORRHOEA	UY54031	Urethritis door gonokokken Y	Male gonococcal urethritis
GONORRHOEA	W6471	Zwangerzap en gonorroe	Pregnancy and gonorrhoea
GONORRHOEA	X098	Infektie gonokokken	Gonococcal infection
GONORRHOEA	X0980	Infektie gonokokken acuut X NNO	Acute female gonococcal infection, not further specified
GONORRHOEA	X09800	Bartholinitis gonokokken acuut	Acute gonococcal bartholinitis
GONORRHOEA	X09802	Vulvovaginitis gonokokken acuut	Acute gonococcal vulvovaginitis
GONORRHOEA	X09815	Cervicitis acuut gonokokken	Acute gonococcal cervicitis
GONORRHOEA	X09816	Endometritis acuut gonokokken	Acute gonococcal endometritis
GONORRHOEA	X09817	Salpingitis acuut gonokokken	Acute gonococcal salpingitis
GONORRHOEA	X09821	Bartholinitis gonokokken chronisch	Chronic gonococcal bartholinitis
GONORRHOEA	X09823	Vulvovaginitis gonokokken chronisch	Chronic gonococcal vulvovaginitis
GONORRHOEA	X09835	Cervicitis gonokokken chronisch	Chronic gonococcal cervicitis
GONORRHOEA	X09836	Endometritis gonokokken chronisch	Chronic gonococcal endometritis
GONORRHOEA	X09837	Salpingitis gonokokken chronisch	Chronic gonococcal salpingitis
GONORRHOEA	X09839	Gonorroe chronisch X NNO	Chronic female gonorrhoea, not further specified
GONORRHOEA	X61614	Vulvovaginitis gonococcen [sic]	Gonococcal vulvovaginitis
GONORRHOEA	XA54011	Cervicitis gonokokken	Gonococcal cervicitis
GONORRHOEA	Y09812	Infektie gonokokken acuut prostaat	Acute gonococcal prostate infection
GONORRHOEA	Y09813	Infektie gonokokken acuut testis	Acute gonococcal testis infection
GONORRHOEA	Y09814	Infektie gonokokken acuut zaadblaasjes	Acute gonococcal infection of the seminal vesicles
GONORRHOEA	Y09819	Infektie gonokokken acuut Y NNO	Acute male gonococcal infection, not further specified
GONORRHOEA	Y0982	Infektie gonokokken chronisch	Chronic gonococcal infection
GONORRHOEA	Y09832	Infektie gonokokken chronisch prostaat	Chronic gonococcal prostate infection
GONORRHOEA	Y09833	Infektie gonokokken chronisch testis	Chronic gonococcal testis infection
GONORRHOEA	Y09834	Infektie gonokokken chronisch zaadblaasjes	Chronic gonococcal infection of seminal vesicles
GONORRHOEA	Y09838	Infektie gonokokken chronisch rectum Y	Chronic male gonococcal rectal infection
GONORRHOEA	Y09839	Infektie gonokokken chronisch Y NNO	Chronic male gonococcal infection, not further specified
			
**SYPHILIS**			
SYPHILIS	A0907	Syfilis congenitaal laat NNO	Late congenital syphilis, not further specified
SYPHILIS	A0909	Syfilis congenitaal NNO	Congenital syphilis, not further specified
SYPHILIS	A0910	Syfilis primair genitaal	Primary genital syphilis
SYPHILIS	A09130	Syfilis secundair + exantheem	Secondary syphilis and exanthema
SYPHILIS	A0958	Syfilis tertiair	Tertiary syphilis
SYPHILIS	A096	Syfilis latent laat	Latent late syphilis
SYPHILIS	A0971	Syfilis serologie positief	Positive syphilis serology
SYPHILIS	A0979	Syfilis NNO	Syphilis, not further specified
SYPHILIS	AA5103	Primaire genitale syfilis	Primary genital syphilis
SYPHILIS	AA5112	Primaire anale syfilis	Primary anal syphilis
SYPHILIS	AA5121	Primair extragenitaal syfilis X	Primary extragenital female syphilis
SYPHILIS	AA528	Syfilis laat latent	Late latent syphilis
SYPHILIS	AA5282	Syfilis laat latent	Late latent syphilis
SYPHILIS	AA5292	Syfilis laat	Late syphilis
SYPHILIS	AA530	Syfilis latent NNO	Latent syphilis, not further specified
SYPHILIS	AA5391	Syfilis	Syphilis
SYPHILIS	AA5392	Syfilis	Syphilis
SYPHILIS	AA65	Endemische syfilis	Endemic syphilis
SYPHILIS	BA51492	Secundaire syfilis	Secondary syphilis
SYPHILIS	K0930	Aneurysma aortae syfilitisch	Syphilitic aortic aneurysm
SYPHILIS	K0931	Aortitis syfilitisch	Syphilitic aortitis
SYPHILIS	K0932	Endocarditis syfilitisch	Syphilitic endocarditis
SYPHILIS	K09381	Pericarditis syfilitisch	Syphilitic pericarditis
SYPHILIS	K09382	Myocarditis syfilitisch	Syphilitic myocarditis
SYPHILIS	KI3983	Endocarditis door syfilis	Syphilitic endocarditis
SYPHILIS	KI4104	Myocarditits door syfilis	Syphilitic myocarditis
SYPHILIS	L09001	Pijn arm NNO [observations to be dropped due to internal Medidoc assignment error]	Arm pain, not further specified
SYPHILIS	L09001	Syfilis congenitaal locomotorisch	Congenital locomotor syphilis
SYPHILIS	L09161	Syfilis secundair bot	Secondary bone syphilis
SYPHILIS	MDC_X70	Syphilis vrouw	Female syphilis
SYPHILIS	MDC_Y70	Syphilis man	Male syphilis
SYPHILIS	N09490	Syphilis gumma CZS	Syphilis gumma central nervous system
SYPHILIS	N09499	Syfilis neurologisch NNO	Neurologic syphilis, not further specified
SYPHILIS	W6470	Zwangerschap en syfilis	Pregnancy and syphilis
SYPHILIS	X0910	Syfilis primair genitaal X	Primary genital female syphilis
SYPHILIS	X0911	Syfilis primair anaal X	Primary anal female syphilis
SYPHILIS	X0913	Syphilis secundair cutaan	Secondary skin syphilis
SYPHILIS	X0919	Syfilis X NNO	Female syphilis, not further specified
SYPHILIS	X092	Syfilis latent X	Latent female syphilis
SYPHILIS	X0958	Syfilis laattijdig tertiair X	Late tertiary female syphilis
SYPHILIS	X096	Syfilis laattijdig latent X	Late latent female syphilis
SYPHILIS	X61652	Ulcus vulva syfilis	Syphilitic vulvar ulcer
SYPHILIS	Y0910	Syfilis sjanker Y	Male syphilitic chancre
SYPHILIS	Y092	Syfilis latent Y	Latent male syphilis
SYPHILIS	YA513	Syfilis secundair mucocutaan Y	Secondary mucocutaneous male syphilis
